# Proper PIN1 Distribution Is Needed for Root Negative Phototropism in *Arabidopsis*

**DOI:** 10.1371/journal.pone.0085720

**Published:** 2014-01-21

**Authors:** Kun-Xiao Zhang, Heng-Hao Xu, Wen Gong, Yan Jin, Ya-Ya Shi, Ting-Ting Yuan, Juan Li, Ying-Tang Lu

**Affiliations:** 1 State Key Laboratory of Hybrid Rice, College of Life Sciences, Wuhan University, Wuhan, China; 2 Institute of Fruit and Tea, Hubei Academy of Agricultural Sciences, Wuhan, China; University of Nottingham, United Kingdom

## Abstract

Plants can be adapted to the changing environments through tropic responses, such as light and gravity. One of them is root negative phototropism, which is needed for root growth and nutrient absorption. Here, we show that the auxin efflux carrier PIN-FORMED (PIN) 1 is involved in asymmetric auxin distribution and root negative phototropism. In darkness, PIN1 is internalized and localized to intracellular compartments; upon blue light illumination, PIN1 relocalize to basal plasma membrane in root stele cells. The shift of PIN1 localization induced by blue light is involved in asymmetric auxin distribution and root negative phototropic response. Both blue-light-induced PIN1 redistribution and root negative phototropism is mediated by a BFA-sensitive trafficking pathway and the activity of PID/PP2A. Our results demonstrate that blue-light-induced PIN1 redistribution participate in asymmetric auxin distribution and root negative phototropism.

## Introduction

Plants are sessile by nature, and can be adapted to the changing environments through tropic responses, such as hypocotyl phototropism and root negative phototropism [1]–[3]. Whereas plant shoots can maximize capture of light source by hypocotyl phototropism, plant roots bend away from light source as root negative phototropic response to avoid the damage of light and other stressful stimulus from the upper layers of soil, and to facilitate water and nutrient absorption from the soil [2].

For the mechanism of tropic responses, a role for differential distribution of auxin was proposed in classical Cholodny-Went theory [4], in which asymmetric auxin distribution leads to unequal growth of two sides of a bending organ. In recent years, it has been reported that an increased *DR5* activity in the shaded side of hypocotyl is required for hypocotyl phototropism [5]–[8]. In contrast, higher *DR5* activity was demonstrated in the illuminated side of roots exposed to unilateral blue light in root negative phototropic response [9]. Notably, the asymmetric auxin distribution during tropic response is mediated by auxin transporters of the AUXIN RESISTANT/LIKE AUXIN RESISTANT, P-GLYCOPROTEIN, and PIN families [10]–[13].

Root negative phototropism and hypocotyl phototropism are specially regulated by blue light receptor PHOT1 [5], [6], [8], [9], [14]–[20], which perceives the blue light signals and translates into the auxin signaling pathway. Recently, it has been reported that the auxin efflux carriers PIN2 and PIN3 are necessary for asymmetric auxin transport and root negative phototropic response [9], [19]. Upon unilateral blue light illumination, the shift of PIN2 localization that is controlled by blue light and BFA sensitive recycling can change the auxin distribution in roots and result in root negative tropic response [19]. However, the polar localization of PIN2 only controls the basipetal flow of auxin to the elongation zone [21], which implies that the lateral auxin flow in the root tip underlying root negative phototropism also needs other auxin transporters. Recently, it has been indicated that unilateral blue light illumination polarizes PIN3 to the outer lateral membrane of columella cells at the illuminated root side, and increase auxin activity at the illuminated side of roots, where auxin promotes growth and causes roots bending away from the light source [9]. Moreover, the blue-light-induced PIN3 polarization in root negative phototropism is mediated by a BFA-sensitive, GNOM-dependent trafficking pathway and the activity of PID/PP2A. Interestingly, the polar distribution of PIN3 for hypocotyl phototropism, hypocotyl gravitropism and root gravitropism is also regulated by a BFA-sensitive trafficking pathway and the activity of PID [6], .

Previous reports show that PIN1 is required for hypocotyl phototropic response [8], [25] and the unilateral blue light illumination can result in PIN1 relocalization in hypocotyl cells in this process [25]. In gain-of-function *PID* mutants, which exhibit a collapsed root phenotype, PIN1 is relocated to the apical plasma membrane (PM) and the auxin gradient is disrupted in the roots [24], [26]. Notably, several different *PP2A* mutants have a similar phenotype as the *PID* gain-of-function mutants [27], indicating the antagonistic regulation of PIN1 polarization by PID and PP2A. Recently, several PID-dependent Ser/Thr phosphorylation sites in PIN1 were identified that are involved in the basal-to-apical PIN1 polarity shift [28], [29]. The shift in PIN1 localization also requires a BFA-sensitive trafficking pathway. BFA, a fungal toxin that inhibits GNOM, causes PIN1 to accumulate in endosomes called BFA compartments [30]–[32]. GNOM, a member of the ARF-GEF (exchange factors for ARF-GTPases) family, is needed for PIN1 recycling from endosomes to the plasma membrane [33].

In this study, we investigate the role of *PIN1*-regulated auxin distribution during root negative phototropic response. Our results show that blue light illumination can shift the PIN1 localization from intracellular compartments to the basal plasma membrane in root stele cells, which result in asymmetric auxin distribution and root negative phototropism. Moreover, the BFA-sensitive vesicle trafficking pathway and the activity of PID/PP2A are also needed for blue-light-induced PIN1 distribution and root negative phototropic response.

## Results

### PIN1 is Needed for Root Negative Phototropism and Asymmetric Auxin Distribution

**Figure 1:**
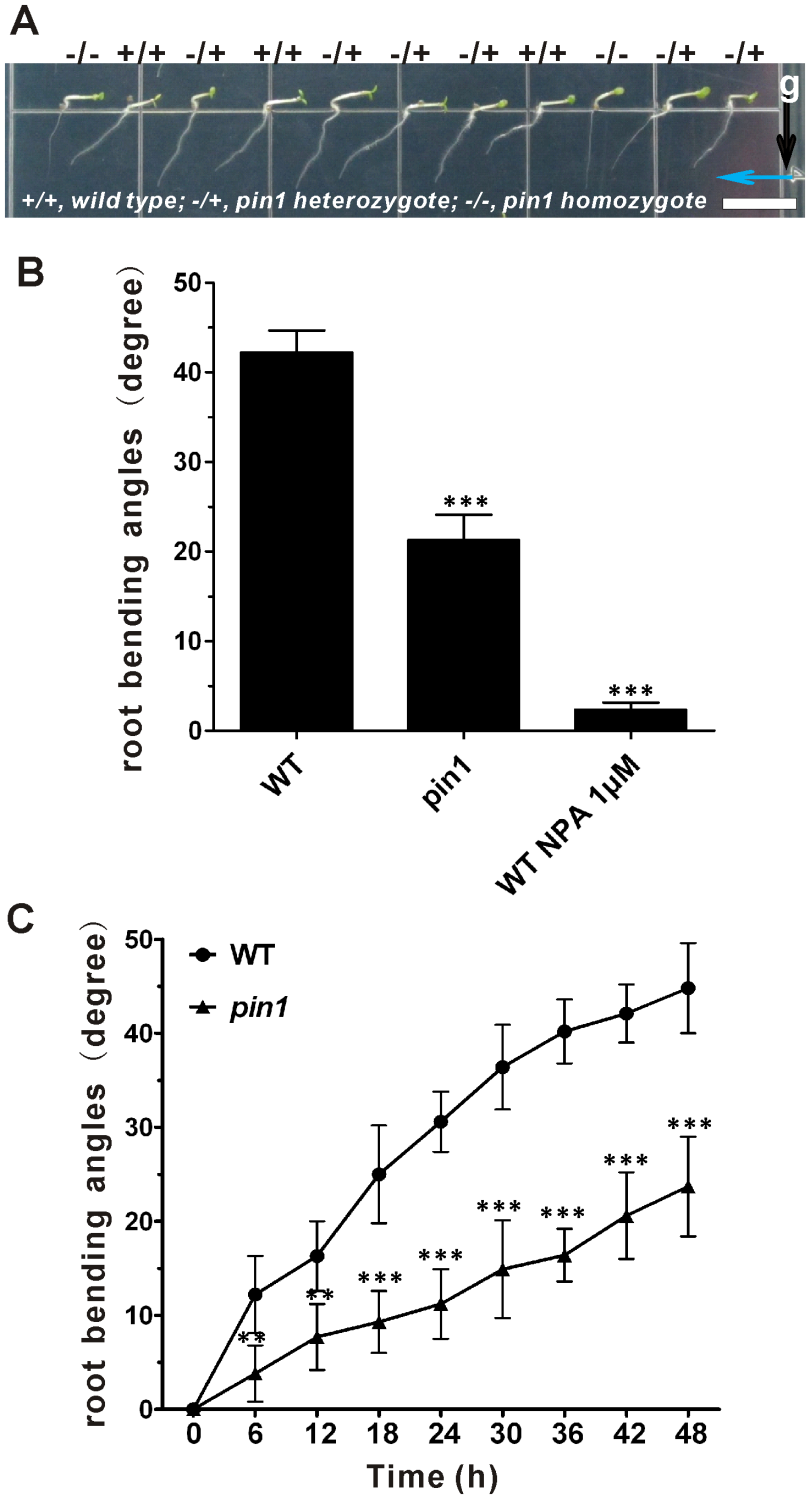
Root negative phototropic response of *pin1* mutant seedlings. (A) Images of 2-day-old etiolated seedlings of the *pin1* homozygous mutants grown on vertical plates, and then exposed to unilateral blue light (10 µmol m^−2^sec^−1^) for another 48 h. For the experiments with *pin1* mutant, the seeds from *pin1* heterozygote plants were used because *pin1* homozygote is infertile. After root bending assays were performed, the seedlings were identified to be wild-type, *pin1* homozygote or heterozygote by PCR. Only data for root bending from the seedlings of *pin1* homozygote were used for statistical analysis. The arrows indicate the direction of blue light (blue) and gravity (black). *+/+*, wild type; *−/+*, *pin1* heterozygote; *1−/−*, *pin1* homozygote**.** Bar = 1 cm. (B) Root bending angles of *pin1* homozygous, WT and NPA-treated WT plants (1 µM). The bending angles of the roots away from the vertical direction were measured after 48 h unilateral blue light illumination and average curvatures were calculated. Values are the average of three biological replicates (n >10 per time point on each replicate). (C) Root bending kinetics of WT, and *pin1* homozygous seedlings. Root curvatures were measured every 6 hours under unilateral blue light illumination and average curvatures were calculated. Values are the average of three biological replicates (n >10 per time point on each replicate). Error bars represent SE and the symbols ** and *** indicate significant difference at P<0.01 or P<0.001 between WT and *pin1* or NPA treated WT in (B) or between WT and *pin1* at each time point in (C), as determined by Student’s *t*-test.

Recently, it has been reported that auxin efflux carriers are involved in plant tropic response, such as hypocotyl phototropism, hypocotyl gravitropism, root gravitropism and root negative phototropism [5], [6], [8], [9], [19], [22], [25]. The reduced root negative phototropic response in *pin3-4* mutant implies the redundant function of auxin transporters in this process [9]. PIN1 as a key factor in hypocotyl phototropism may also participate in root negative phototropism. In order to test the contribution of PIN1 to the root negative phototropism, we used the loss-of-function *pin1* in the following experiments. Because *pin1* null mutants are completely sterile [34], *PIN1*/*pin1* heterozygous seedlings were used for the physiological experiments and *pin1* homozygous plants were identified by genotyping after the experiments. As expected, the detailed kinetics of root negative phototropic bending in *pin1* homozygous mutants confirmed that PIN1 is involved in root negative phototropic response (Fig. 1A–1C). Furthermore, the effects of polar auxin transport inhibitor N-1-naphthylphthalamic acid (NPA) in wild-type seedlings (Fig. 1B) indicated that polar auxin transport is needed for root negative phototropic response.

**Figure 2:**
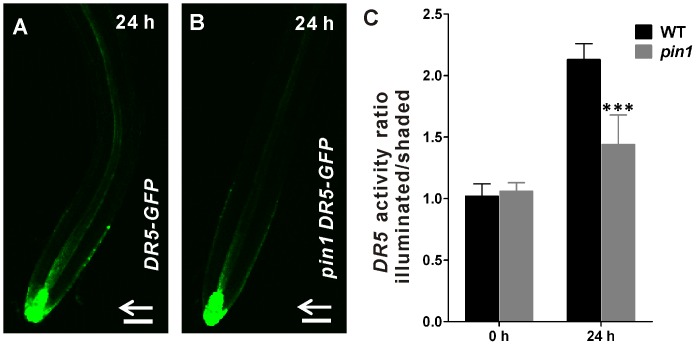
PIN1 activity is needed for asymmetric auxin distribution in root negative phototropism. (A–B) *DR5* activity was monitored in the *DR5_REV_::GFP* (A) and *pin1 DR5_REV_::GFP* (B) seedlings exposed to unilateral blue light illumination (10 µmol m^−2^sec^−1^) for 24 h. Arrows indicate blue light direction. Bars = 50 µm. (C) GFP signal intensities in (A–B) were quantified and their ratios at the illuminated side versus the shaded side are presented in (C). At least twelve seedlings were imaged per line for each of three replicates. Error bars represent standard deviation and *** indicate significant difference at P<0.001, as determined by Student’s *t*-test.

Next, we investigated whether PIN1 is needed for generating the asymmetric auxin distribution in root negative phototropic response. We used the auxin responsive *DR5_REV_::GFP* line, which reliably reveals the pattern of auxin distribution in roots [35]. Consistent with previous report [9], an asymmetric *DR5* activity is detected in unilateral blue light illuminated roots of wild-type seedlings (Fig. 2A). In contrast, reduced asymmetry in *DR5* activity is observed in *pin1* homozygous mutants as compared with the wild-type plants (Fig. 2A–2C). These results reveal a role of PIN1 in root negative phototropism, and indicate that PIN1 activity is needed for the generation of asymmetric auxin distribution during root negative phototropic response.

### The Shift of PIN1 Localization is Regulated by Blue Light

**Figure 3:**
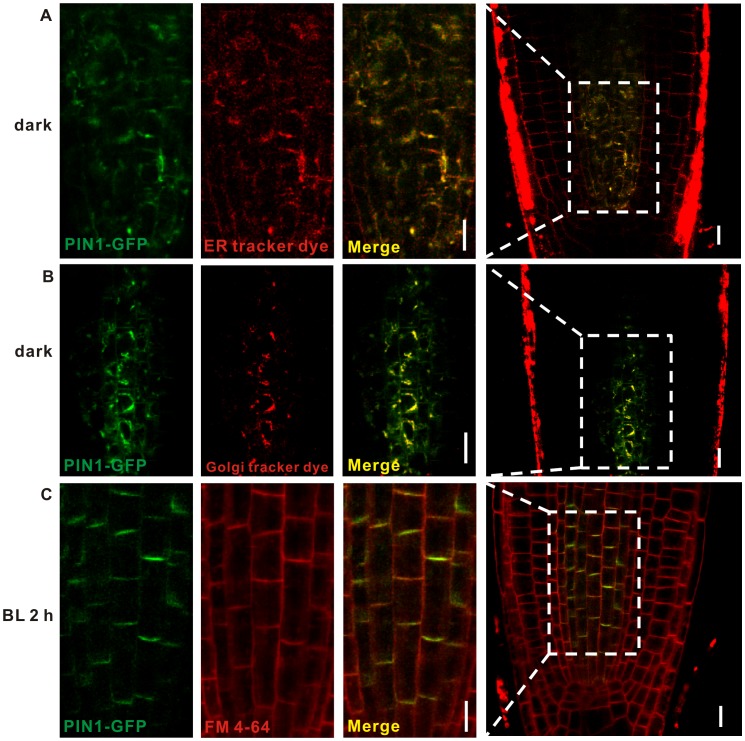
Subcellular localization of PIN1 in the roots of plants grown in the dark or exposed to blue light. (A–B) Co-localization (yellow) of PIN1-GFP (green) with ER or Golgi tracker dye (red) in the dark. Four-day-old etiolated seedlings of the *PIN1::PIN1-GFP* (green) marker line were pretreated with either ER tracker dye (A) or Golgi tracker dye (B) for 30 minutes in the dark before imaging. (C) Co-localization of PIN1-GFP (green) with FM 4–64 (red) after 2 h of blue light illumination. Four-day-old etiolated seedlings of the *PIN1::PIN1-GFP* marker line were exposed to blue light (10 µmol m^−2^sec^−1^) for 2 h, and treated with FM 4–64 for 10 minutes before imaging. (A–C) At least twelve seedlings were imaged per line for each of three replicates. Bars = 10 µm. Panels in the left are enlargements of the boxed regions shown in the rightmost column.

The directional flow of auxin is mediated by auxin polar transporters of the AUX and PIN families [10], [11], [13], and PIN1 is key factor in the asymmetric distribution of auxin during hypocotyl phototropism. The polarity of the subcellular localization of PIN1 has been shown to determine the acropetal flow of auxin and thereby regulate auxin redistribution in roots [36]. Thus, we analyzed the subcellular localization of PIN1 under the blue light illumination using the *PIN1::PIN1-GFP* marker line. In darkness, PIN1-GFP was internalized and lost from the plasma membrane (PM) in the root stele cells (Fig. 3A, and 3B). Endoplasmic reticulum (ER) and Golgi tracker dye staining indicated that PIN1-GFP localized to the ER and Golgi (Fig. 3A and 3B), in addition to the vacuole in the dark [37], [38]. However, upon unilateral blue light illumination, PIN1-GFP relocalized to the basal plasma membrane, as determined by co-localization with the FM 4–64 membrane stain (Fig. 3C). We also assayed the effect of blue light on PIN1 distribution in the roots of wild-type plants. After 15 minutes of blue light illumination, some PIN1-GFP protein began to localize to the basal PM of root stele cells, and PIN1-GFP continued to relocate to the basal PM for up to 2 h of light treatment, suggesting that blue light induces the redistribution of PIN1 (Fig. S1A–1D).

**Figure 4:**
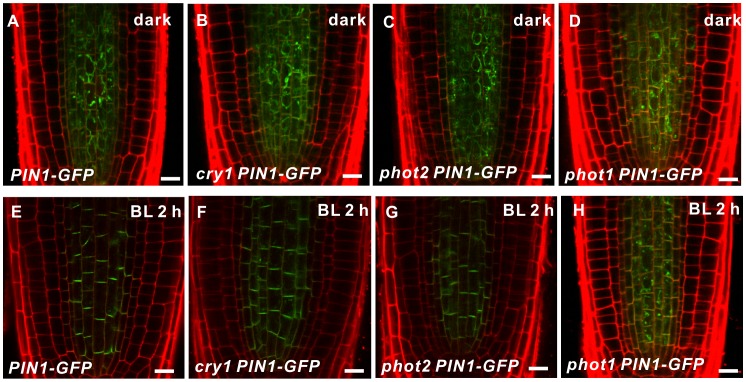
Blue-light-induced PIN1 is regulated by phot1 during root negative phototropism. (A–H) PIN1 localization, as revealed by GFP fluorescence, in the stele of *PIN1::PIN1-GFP* (A, E), *PIN1::PIN1-GFP cry1* (B, F), *PIN1::PIN1-GFP phot2* (C, G), and *PIN1::PIN1-GFP phot1* (D, H) seedlings grown in darkness (A–D) or under blue light (E–H) (10 µmol m^−2^sec^−1^) for 2 h. At least twelve seedlings were imaged per line for each of three replicates. Bars = 10 µm.

Both phototropin and cryptochrome are blue-light receptor families in *Arabidopsis*. To test whether these blue light receptors are involved in PIN1 redistribution, *cry1, phot2,* and *phot1* were crossed with the *PIN1::PIN1-GFP* line and the subcellular localization of PIN1-GFP was observed in the resulting progeny. When these progenies were treated with unilateral blue light, the roots of *cry1* and *phot2* plants, exhibit a normal root negative phototropic response [9] and normal distribution of PIN1-GFP at the basal PM of stele cells (Fig. 4A–4C, and 4E–4G). However, in *phot1* mutant, PIN1-GFP was still internalized and no visible effect on blue-light-induced PIN1 redistribution was detected in the roots, even after 2 h of unilateral blue light illumination (Fig. 4D, and 4H). Therefore, the blue-light-induced PIN1 redistribution is regulated by the blue-light receptor PHOT1.

### Blue-Light-Induced PIN1 Redistribution is Regulated by a BFA-sensitive, GNOM-dependent Trafficking Pathway

The blue-light-induced PIN1 redistribution in root negative phototropism can result from *de novo* protein synthesis or degradation. To investigate whether protein synthesis is involved in blue-light-induced PIN1 redistribution in root negative phototropism, we used the protein synthesis inhibitor cycloheximide (CHX). Four-day-old etiolated wild-type seedlings were pretreated with CHX for 1 h in the dark, and then exposed to blue light illumination for 2 h. Our results showed that the blue-light-induced PIN1 redistribution occurred normally in *PIN1::PIN1-GFP* plants treated with CHX (Fig. S2A). These results suggest that *de novo* protein synthesis is not involved in the blue-light-induced PIN1 redistribution. In addition, the possible role of proteolytic protein degradation in this process was analyzed using MG132, an inhibitor of the 26S proteasome. No visible effect on blue-light-induced PIN1 redistribution was observed by using MG132 treatment (Fig. S2B). Furthermore, *snx1* mutants, defective in PIN2 degradation [39], [40], showed normal PIN1 localization and root negative phototropic response under blue light illumination (Fig. S2C, and 2D). These results indicated that protein degradation does not participate in blue-light-induced PIN1 redistribution in root negative phototropic response.

**Figure 5:**
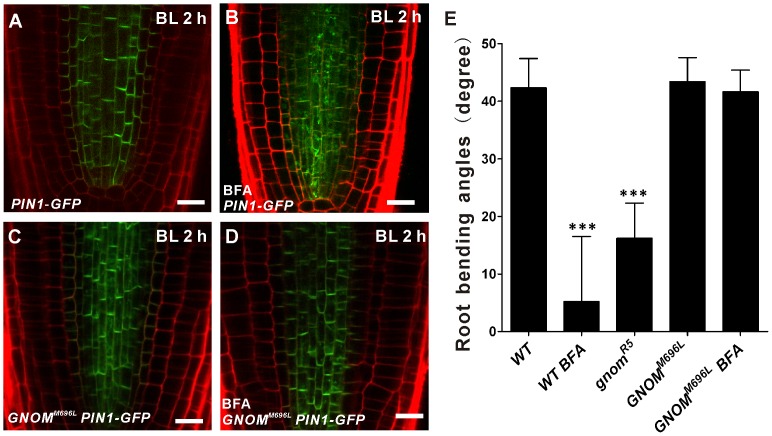
Involvement of the BFA-sensitive, GNOM-dependent trafficking pathway in blue-light-induced PIN1 distribution. (A–D) PIN1 localization, as revealed by GFP fluorescence, was examined in the steles of *PIN1::PIN1-GFP* (A or C) and *PIN1::PIN1-GFP GNOM^M696L^* (B or D) seedlings treated or not with BFA and exposed to unilateral blue light for 2 h. Four-day-old etiolated seedlings were pretreated with DMSO as a control or BFA (50 µM) in the dark for 1 h, and subsequently exposed to unilateral blue light illumination (10 µmol m^−2^sec^−1^) for 2 h. At least twelve seedlings were imaged per line for each of three replicates. Arrows indicate blue light direction. Bars = 10 µm. (E) Root bending angles under BFA treatment. The bending angles of the roots away from the vertical direction were measured after 48 h unilateral blue light illumination and average curvatures were calculated. Values are the average of three biological replicates (n >10 per time point on each replicate). Error bars represent SE and *** indicate significant difference at P<0.001, as determined by Student’s *t*-test.

PIN proteins are recycled constitutively between endosomes and the plasma membrane, and the recycling is sensitive to the vesicle trafficking inhibitor BFA [30], [31]. Thus, the BFA-sensitive, vesicle trafficking pathway may be involved in blue light-induced PIN1 redistribution in root negative phototropism. To explore this possibility, BFA was used to test whether the BFA-sensitive trafficking pathway is involved in blue-light-induced PIN1 redistribution in root negative phototropism. BFA treatment strongly inhibited the shift localization of PIN1 from intracellular compartments to basal plasma membrane (Fig. 5A, and 5B). Furthermore, previous reports also demonstrated that the root negative phototropic response is affected by BFA treatment [9], [19]. These results demonstrate that the BFA-sensitive trafficking pathway is involved in blue-light-induced PIN1 redistribution and root negative phototropism.

GNOM has been reported to mediate PIN proteins recycling to the plasma membrane and is inhibited by BFA [33]. To test whether GNOM is involved in BFA-sensitive vesicle trafficking pathway for blue-light-induced PIN1 redistribution, the *GNOM^M696L^* lines that express a genetically engineered BFA-resistant version of GNOM were used [33]. In *GNOM^M696L^* roots, no visible differences on blue-light-induced PIN1 redistribution and root negative phototropic responses were detected in both the presence and absence of BFA (Fig. 5C, and 5D) [9]. In addition, it also has been shown that the partial loss-of-function *gnom^R5^* mutants exhibit the reduced root negative phototropic response [9]. These results suggested that blue-light-induced PIN1 redistribution is regulated by BFA-sensitive, GNOM-dependent trafficking pathway.

### Blue-Light-Induced PIN1 Distribution and Root Negative Phototropism are Mediated by *PID/PP2A*

Given that the shift in PIN1 polarity is mediated by the antagonistic PID/PP2A phosphorylation pathway [27], and that PID/PP2A-dependent PIN3 polarization is involved in root negative phototropism [9], the polar distribution of PIN1 in blue-light-induced root negative phototropic response may be also modulated by this pathway.

**Figure 6:**
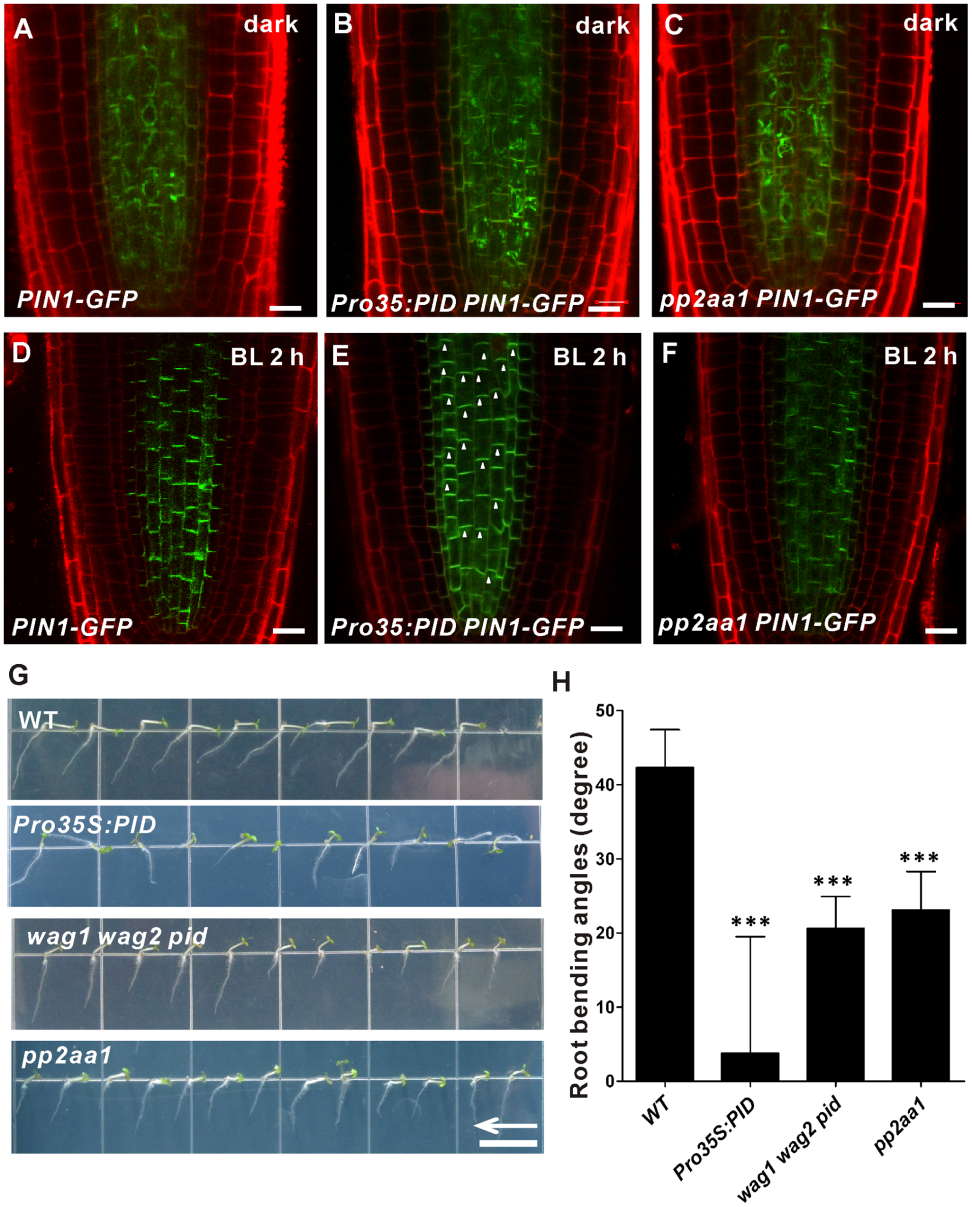
Blue-light-induced PIN1 distribution is mediated by PID/PP2A. (A–F) PIN1 localization, as revealed by GFP fluorescence, in the steles of 4-day-old etiolated *PIN1::PIN1-GFP* (A, D), *PIN1::PIN1-GFP Pro35S:PID* (B, D), and *PIN1::PIN1-GFP pp2aa1* (C, F) seedlings grown in darkness (A–C) or under blue light (D–F) (10 µmol m^−2^sec^−1^) for 2 h. At least twelve seedlings were imaged per line for each of three replicates. Arrowheads show the apical plasma membrane localization of PIN1 in *Pro35S:PID* seedlings under blue light illumination. Arrows indicate blue light direction. Bars = 20 µm. (G) Images of 2-day-old etiolated seedlings of the wild-type, *Pro35S:PID*, *wag1 wag2 pid* and *pp2aa1* mutants grown on vertical plates, and then exposed to unilateral blue light (10 µmol m^−2^sec^−1^) for another 48 h. The arrows indicate the direction of blue light. Bar = 1 cm. (H) Root bending angles of mutants. The bending angles of the roots away from the vertical direction were measured after 48 h unilateral blue light illumination and average curvatures were calculated. Values are the average of three biological replicates (n >10 per time point on each replicate). Error bars represent SE and *** indicate significant differen**c**e at P<0.001, as determined by Student’s *t*-test.

To test this hypothesis, we first examined the effect of PID on blue-light-induced PIN1 redistribution in root negative phototropism. Thus, *Pro35S:PID* seedlings constitutively expressing PID [26] were used. In the dark, the localization of PIN1-GFP was the same as in the wild type (Fig. 6A, and 6B). Upon unilateral blue light illumination, in the illuminated roots of *Pro35S:PID* plants, which had severe defects in root negative phototropism [9], most of the PIN1-GFP localized to the apical plasma membrane (Fig. 6E). Given that WAG1 and WAG2 are the closest homologues of PID, the triple mutant *wag1 wag2 pid* was used for phenotype analyses [41], [42]. This triple mutant *wag1 wag2 pid* showed the reduced root negative phototropic response (Fig. 6G, and 6H), consistent with a previous report [9]. These results suggest that the PID-mediated pathway is involved in blue-light-induced PIN1 redistribution during root negative phototropism.

PP2A phosphatase is also an important regulator of PIN1 apical-basal targeting and auxin distribution [27]. To explore the possible role of *PP2A* in blue-light-induced PIN1 redistribution, the *pp2aa1* (*rcn1*) mutant that lacks the phosphatase activity of PP2AA1 was used. In the dark, PIN1-GFP localization was the same in the *pp2aa1* mutant and wild type (Fig. 6A, and 6C). However, upon illumination, only some of the PIN1-GFP became polarized to the basal PM in *pp2aa1* seedlings (Fig. 6D, and 6F). Combined with our previous observation that *pp2aa1* had a reduced root negative phototropic response [9], these results demonstrate that PP2A activity is involved in blue-light-induced PIN1 redistribution during root negative phototropism.

## Discussion

The classical Cholodny-Went theory [4] states that tropic responses are due to the asymmetric distribution of the growth regulator auxin. Recently, studies using the auxin response reporter *DR5* demonstrate that increased *DR5* activity on the shaded side of the hypocotyl is required for hypocotyl phototropism [5]–[7]. In this study, we showed that *DR5* activity is increased at the illuminated side of roots exposed to unilateral blue light (Fig. 2A) [9]. Moreover, we found that PIN1 is necessary for the generation of asymmetric auxin distribution and root negative phototropic response. Upon blue light illumination, blue light receptor PHOT1 modulates the expression of *PID* and *PP2A.* Furthermore, PID and PP2A antagonistically regulate the polar targeting of PIN1. The basal plasma membrane localization of PIN1 in root stele directs the acropetal flow of auxin to root tip [36]. In addition, blue light induces the asymmetric distribution of PIN3 at the outer lateral membrane of columella cells illuminated with unilateral blue light, resulting in the flow of auxin to the illuminated side of roots [9]. Then, blue-light-induced apical PM localization of PIN2 in the epidermis cells of the root [19], [40], [43] directs the basipetal flow of auxin to the elongation zone [21]. The resultant asymmetric distribution of auxin promotes differential growth between the shaded and illuminated side of roots, resulting in root negative phototropism (Fig. S3).

Our data indicate that the blue-light-induced PIN1 distribution, which is regulated by the activity of PID and PP2AA1, is essential for root negative phototropism. It has been reported that PID/PP2A antagonistically mediate PIN1 localization [24], [27]–[29], [32], [44]. In these reports, the phosphorylation status of PIN1 have been shown to alter its polar localization in response to different environmental and endogenous cues [24], [27]–[29], [32], [44]. While PID kinase can directly phosphorylate PIN1 to promote the apical localization of PIN1, PP2A phosphatase operates antagonistically to promote the basal localization of PIN1 in the embryo and the root [27]. The PID-dependent PIN1 polarization is regulated by a BFA-sensitive, GNOM-dependent trafficking pathway during organogenesis and development [32], [42], [45]. Furthermore, blue-light-induced PIN2 distribution is regulated by a BFA-sensitive trafficking pathway during root negative phototropism (Wan et al. 2012), and blue-light-induced PIN3 polarization is regulated by a BFA-sensitive, GNOM-dependent trafficking pathway during root negative phototropic response [9]. Thus, these data suggest that PID/PP2A-mediated PIN1 polarization via a BFA-sensitive, GNOM-dependent trafficking pathway is a universal mechanism to direct polar auxin transport in response to environmental and endogenous cues.

It has been reported that blue light can enhance the expression of *DR5* activity in root stele (Wan et al. 2012). The increased auxin accumulation in root stele should be transported to the root tip, where the blue-light-induced PIN3 polarization can control the auxin flow to the illuminated side of roots [9]. Based on the analyses of the *pin1* mutant, our results also indicated that PIN1 is involved in root negative phototropism. Notably, we also showed that blue light illumination can regulate the shift of PIN1 from intracellular compartments to basal plasma membrane in root stele cells, which is essential for the acropetal transport of auxin to increae auxin accumulation in the root tip. Thus, the blue-light-induced PIN1 redistribution in root stele cells is needed for the asymmetric auxin distribution during root negative phototropic response.

In this report, our mutant analyses revealed that *PID* and *PP2A* are essential for root negative phototropic response. Both the *PID* over-expression line and the *pp2aa1* mutant can disturb PIN1 polar targeting in root stele cells with abnormal root negative phototropic response. Also, other reports indicate that PID and PP2A can antagonistically regulate PIN1 phosphorylation to direct auxin flux and that PID-dependent phosphorylation pathway is needed for PIN3 polarization in hypocotyl phototropism [6], [27]. However, it has been reported that *PID*, as well as *WAG1* and *WAG2*, is not expressed in root stele [42], implying that PID cannot regulate PIN1 polar targeting through physical interaction. Thus, how these kinases modulate PIN1 distribution in root stele remains unknown in root negative phototropic response.

Increased auxin activity at the illuminated side of roots is expected to induce the expression of auxin response factors that promote asymmetric growth and root bending away from the light source. *ARF7/NPH4*, an auxin response factor, has been reported to be involved in hypocotyl phototropism [46], [47]. As expected, we found that the *arf7-1* mutant exhibits a reduced root negative phototropic response (Fig. S4A and B), indicating the involvement of *ARF7* in this process.

## Materials and Methods

### Plant Material

The following published transgenic and mutant lines were used in this study: *DR5_REV_:GFP* [35]; *PIN1:PIN1-GFP* [36]; *GNOM^M696L^* [33]; *pin1* [8]; *Pro35S:PID* [26]; *pp2aa1* [48]; *cry1* (SALK_069292); *phot1* (SALK_146058); *phot2* (SALK_142275); *wag1 wag2 pid* [42]; and *arf7-1* (SALK_040394). The double or triple mutants and/or transgenic lines were obtained by crossing the respective lines above, were confirmed by PCR and are available upon request. All PCR primers used for genotyping are listed in Table S1.

### Plant Growth and Light Conditions

*Arabidopsis thaliana* seeds were surface sterilized with 5% bleach for 5 min, washed three times with sterile water, and plated on agar medium containing half-strength Murashige and Skoog medium (1962) and 0.8% agar (w/v). Seedlings were grown in darkness for 2–4 days at 22°C and stimulated by blue light for further analysis.

The blue light (nm = 475) was provided by an R30 LED Light (enLux) and LH-100SP-LED (NK system). Light fluence rates were measured by a Li250 quantum photometer (Li-Cor, www.licor.com). All experiments in darkness were carried out under a dim green safe light.

### Measurement of Root Negative Phototropism

*Arabidopsis thaliana* seeds were grown in the dark for 2 days and then transferred to unilateral blue light illumination for 2 days as described previously [14]. Root bending angles were analyzed using Image J software (http://rsb.info.nih.gov/ij/) and plotted using Prism 5.0 software (GraphPad, www.graphpad.com). At least three independent experiments were carried out.

### Pharmacological Treatments

Four-day-old etiolated seedlings on half-strength Murashige and Skoog medium were treated with brefeldin A (BFA; 50 µM), cycloheximide (CHX; 50 µM), or carbobenzoxy-Leucyl-Leucyl-leucinal (MG132; 50 µM) for 1 h in the dark. The seedlings were then exposed to unilateral blue light and imaged. In experiments involving the phototropic response, 2-day-old etiolated seedlings grown on plates without drug treatment were transferred to solid half-strength Murashige and Skoog medium containing NPA (1 µM) and exposed to unilateral blue light for 2 days. In control experiments, seedlings were treated with an equal amount of solvent (DMSO). Propidium iodide (PI; 0.05%) was dissolved in distilled water. ER tracker dye (1 µM), Golgi tracker dye (330 µg/ml), and FM 4–64 (5 µM) were used to define the localization of PIN1-GFP. Each experiment was performed at least three times.

### Confocal Microscopy

An Olympus (www.olympus.com) FV1000 ASW confocal scanning microscope was used. Emission wavelengths were as follows: PI, 600 to 640 nm; FM 4–64, 600 to 700 nm and GFP, 500 to 540 nm. Four-day-old etiolated seedlings were grown in the dark and stimulated with unilateral blue light. Etiolated seedlings were placed on slides and shoots were removed with a blade, leaving only the roots for quick confocal observations. The signal intensity was measured using Photoshop CS4 and Image J software (http://rsb.info.nih.gov/ij/). The fluorescence intensity ratios were obtained by comparing DR5-GFP fluorescence intensities between the illuminated side and shaded side of the root in the responsive part. At least twelve seedlings were imaged per line for each of three replicates.

## Supporting Information

Figure S1 **The effect of blue light on PIN1 distribution over time.** (A–D) PIN1 localization, as revealed by GFP fluorescence, in the root stele cells of *PIN1::PIN1-GFP* plants grown in darkness (A) and then exposed to unilateral blue light (10 µmol m^−2^sec^−1^) for 15 min (B), 30 min (C) or 2 h (D).
(TIF)

Figure S2 **The** ***de novo*** **protein synthesis and degradation are not involved in root negative phototropism.** (A–C) PIN1 localization, as revealed by GFP fluorescence, in the stele of *PIN1::PIN1-GFP* plants treated with CHX (A), MG132 (B) or *snx1 PIN1::PIN1-GFP* mutants (C). Four-day-old etiolated seedlings of the *ProPIN1:PIN1-GFP* marker line were pretreated with CHX (50 µM) or MG132 (50 µM) in the dark for 1 h, and then subsequently exposed to unilateral blue light illumination (10 µmol m^−2^sec^−1^) for 2 h. Bars = 10 µm. (D) Root bending angles of wild-type and *snx1* mutants. The bending angles of the roots away from the vertical direction were measured after 48 h unilateral blue light illumination (10 µmol m^−2^sec^−1^) and average curvatures were calculated. Values are the average of three biological replicates (n >10 per time point on each replicate). Error bars represent SE.
(TIF)

Figure S3 **Model for root negative phototropism.** Based on the model for hypocotyl phototropism [6], the regulation pathway of root negative phototropism is summarized in Fig. S3. Upon blue light illumination, blue light receptor PHOT1 modulates the expression of *PID* and *PP2A.* Furthermore, PID and PP2A antagonistically regulate the polar targeting of PIN1. The basal plasma membrane localization of PIN1 in root stele directs the acropetal flow of auxin to root tip. In addition, blue light induces the asymmetric distribution of PIN3 at the outer lateral membrane of columella cells illuminated with unilateral blue light, resulting in the flow of auxin to the illuminated side of roots. Then, blue-light-induced apical PM localization of PIN2 in the epidermis cells of the root directs the basipetal flow of auxin to the elongation zone. The resultant asymmetric distribution of auxin promotes differential growth between the shaded and illuminated side of roots, resulting in root negative phototropism.
(TIF)

Figure S4 **NPH4/ARF7 is involved in root negative phototropism.** (A–B) The phenotypes (A) and root bending angles (B) in the wild-type and *arf7-1* mutant. Two-day-old etiolated seedlings of the wild-type and *arf7-1* mutant were exposed to unilateral blue light illumination (10 µmol m^−2^sec^−1^) for 2 days. Values are the average of three biological replicates (n >10 per time point on each replicate). The arrows indicate the direction of blue light (blue) and gravity (black). Error bars represent SE and *** indicate significant difference at p<0.001, as determined by Student’s *t*-test. Bar = 0.5 cm.
(TIF)

Table S1 **Primers for genotyping analysis.**
(DOC)
