# Supplementary figures and images for: Proper PIN1 Distribution Is Needed for Root Negative Phototropism in Arabidopsis
Source: PLoS One. 2014 Jan 21;9(1):e85720. doi: 10.1371/journal.pone.0085720 (PMC3897508; doi:10.1371/journal.pone.0085720)

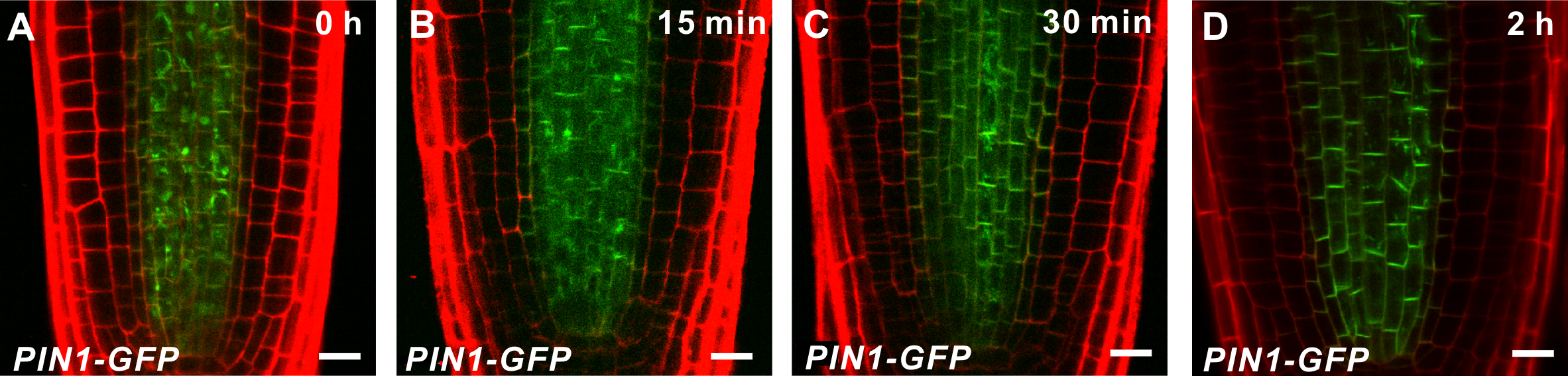

Supplement: Figure S1 — The effect of blue light on PIN1 distribution over time. (A–D) PIN1 localization, as revealed by GFP fluorescence, in the root stele cells of PIN1::PIN1-GFP plants grown in darkness (A) and then exposed to unilateral blue light (10 µmol m−2sec−1) for 15 min (B), 30 min (C) or 2 h (D). (TIF) [file pone.0085720.s001.tif]

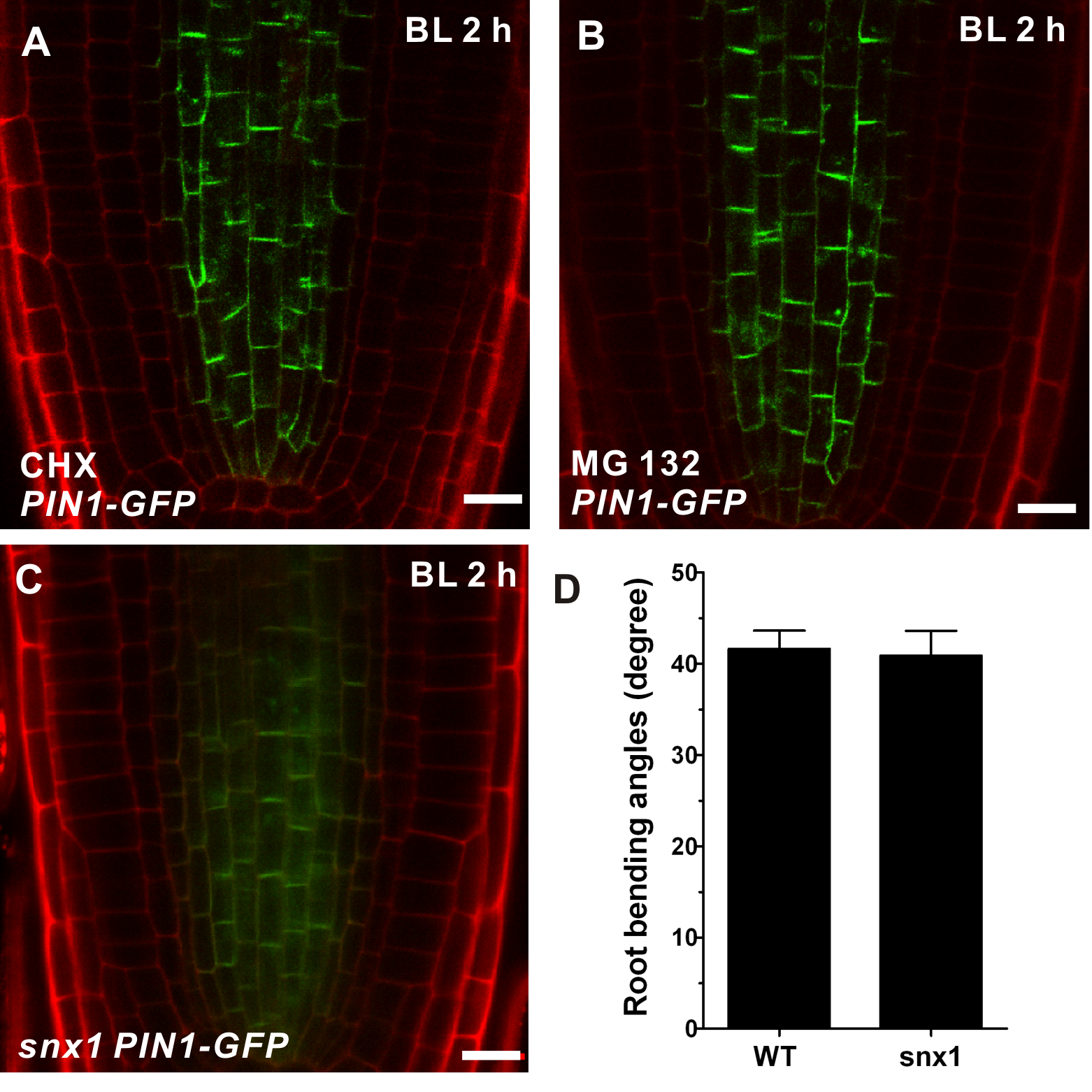

Supplement: Figure S2 — The de novo protein synthesis and degradation are not involved in root negative phototropism. (A–C) PIN1 localization, as revealed by GFP fluorescence, in the stele of PIN1::PIN1-GFP plants treated with CHX (A), MG132 (B) or snx1 PIN1::PIN1-GFP mutants (C). Four-day-old etiolated seedlings of the ProPIN1:PIN1-GFP marker line were pretreated with CHX (50 µM) or MG132 (50 µM) in the dark for 1 h, and then subsequently exposed to unilateral blue light illumination (10 µmol m−2sec−1) for 2 h. Bars = 10 µm. (D) Root bending angles of wild-type and snx1 mutants. The bending angles of the roots away from the vertical direction were measured after 48 h unilateral blue light illumination (10 µmol m−2sec−1) and average curvatures were calculated. Values are the average of three biological replicates (n >10 per time point on each replicate). Error bars represent SE. (TIF) [file pone.0085720.s002.tif]

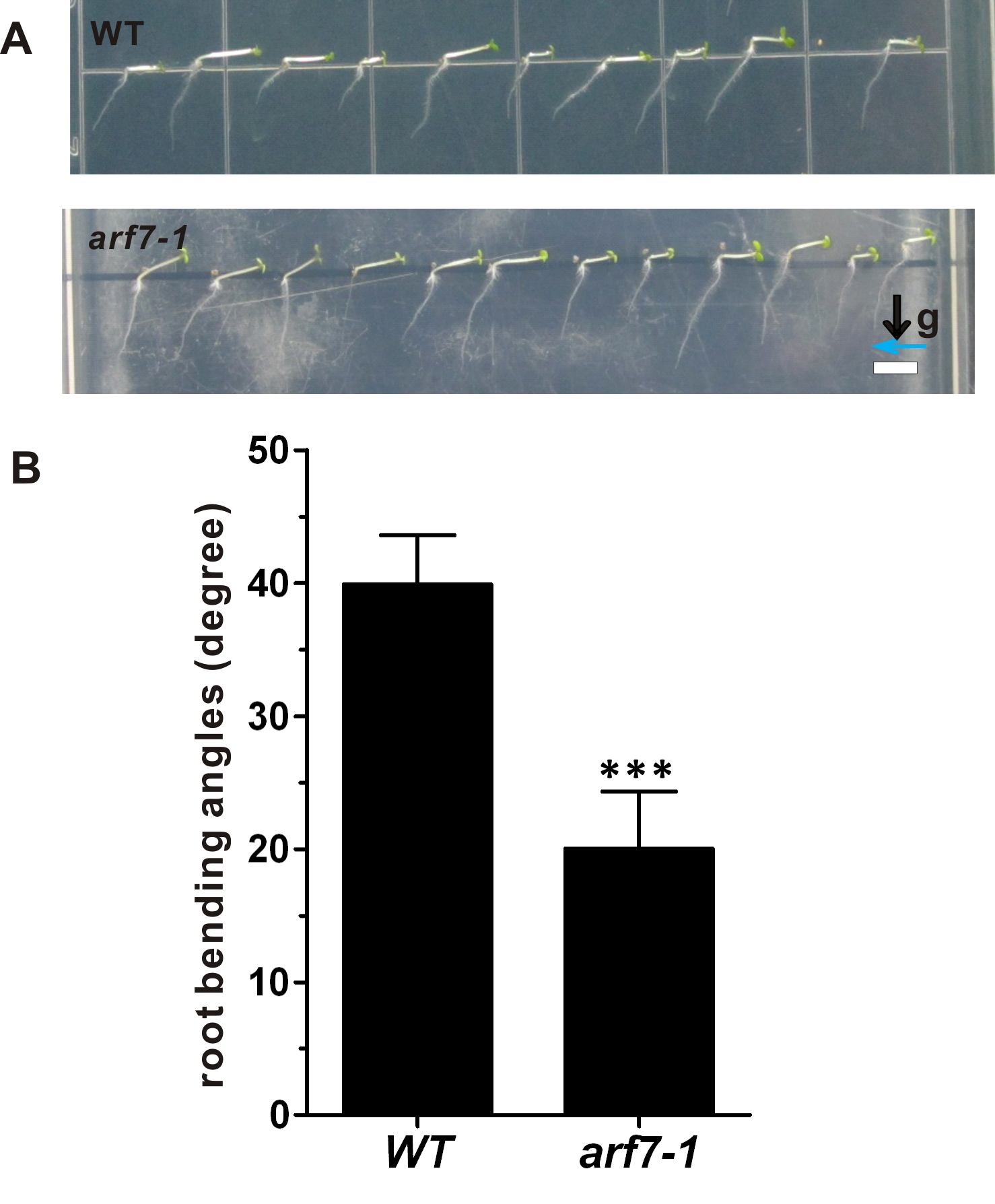

Supplement: Figure S4 — NPH4/ARF7 is involved in root negative phototropism. (A–B) The phenotypes (A) and root bending angles (B) in the wild-type and arf7-1 mutant. Two-day-old etiolated seedlings of the wild-type and arf7-1 mutant were exposed to unilateral blue light illumination (10 µmol m−2sec−1) for 2 days. Values are the average of three biological replicates (n >10 per time point on each replicate). The arrows indicate the direction of blue light (blue) and gravity (black). Error bars represent SE and *** indicate significant difference at p<0.001, as determined by Student’s t-test. Bar = 0.5 cm. (TIF) [file pone.0085720.s004.tif]
