# Supplementary material for: Proper PIN1 Distribution Is Needed for Root Negative Phototropism in Arabidopsis
Source: PLoS One. 2014 Jan 21;9(1):e85720. doi: 10.1371/journal.pone.0085720 (PMC3897508; doi:10.1371/journal.pone.0085720)
Supplement: Table S1 — Primers for genotyping analysis. (DOC) [file pone.0085720.s005.doc]

**Table S1. Primers for genotyping analysis.**

**Primers for genotyping:**

mutants LP RP

| *phot1* | *TCATCACTGATCCTAGGCTTCC* | *GCATCAGGAAGTTCTCGAAC* |
| --- | --- | --- |
| *phot2* | *CGAACCTACAGTTGTTGTGTCTG* | *TGCTACTTCAACCTGCATCC* |
| *cry1* | *TCATGCCACTTGGTTAGACC* | *CGACAGACTGGATACATCATC* |
| *pin1*  *snx1*  *pp2aa1*  LBa1  LBb1.3 | *TGCCACCTTCAATTCAAAAAC*  *CAAGTTTGGGAAGAAGGGATC*  *CAAGGATGTTCAAAGAGCAGC*  *TGGTTCACGTAGTGGGCCATCG*  *ATTTTGCCGATTTCGGAAC* | *TGATTTTCTTGAGACCGATGC*  *CCTTTTTGCAAGCAGATGAAG*  *GATCGAGTTCAAACGAAGCTG* |
